# Long-Term Stable 1-butyl-3-methylimidazolium Hexafluorophosphate/Ag Metal Composite Membranes for Facilitated Olefin Transport

**DOI:** 10.3390/membranes10080191

**Published:** 2020-08-18

**Authors:** Sang Wook Kang

**Affiliations:** 1 Department of Chemistry, Sangmyung University, Seoul 03016, Korea; swkang@smu.ac.kr; Tel.: +82-2-2287-5362; 2 Department of Chemistry and Energy Engineering, Sangmyung University, Seoul 03016, Korea

**Keywords:** olefin, paraffin, ionic liquid, facilitated transport, membranes

## Abstract

For the preparation of long-term stable ionic liquid/Ag nanoparticles composites, we compared the separation performance of 1-butyl-3-methylimidazolium tetrafluoroborate (BMIM^+^BF_4_^−^)/Ag, and 1-butyl-3-methylimidazolium hexafluorophosphate (BMIM^+^PF_6_^−^)/Ag composite membranes with time. Separation performance showed that the BMIM^+^PF_6_^−^/Ag metal composite membrane was more stable than the BMIM^+^BF_4_^−^/Ag metal composite membrane for more than 160 h. These differences in long-term stability in BMIM^+^PF_6_^−^/Ag and BMIM^+^BF_4_^−^/Ag metal composite membranes was attributable to the phase separation between ionic liquid and nanoparticles. In particular, the phase separation between ionic liquid and silver nanoparticles was not observed with time in hydrophobic ionic liquid BMIM^+^PF_6_^−^, confirmed by X-ray photoelectron spectroscopy.

## 1. Introduction

Silver ions can be reversibly reacted with olefin molecules, forming a silver-olefin complex [1,2,3,4,5,6,7,8,9]. Thus, the silver polymer electrolytes have been investigated as an alternative method in the distillation process, requiring high cost and space [2,4]. For example, the facilitated transport of propylene and ethylene through silver polymer electrolyte membranes consisting of AgBF_4_ or AgCF_3_SO_3_ dissolved in either poly(2-ethyl-2-oxazoline) (POZ), poly(N-vinyl pyrrolidone) (PVP) and poly(ethylene oxide) (PEO) showed the separation performance of 50 (propylene/propane) in selectivity and 12 GPU ((1 GPU = 1 × 10^−6^ cm^3^ (STP)/(cm^2^ s cmHg)) in permeance [5]. However, since these metal ion-based systems could be poisoned by C_2_H_2_, CO, and H_2_S, silver ions were limited in application to the industry field [10]. Furthermore, M. McKenna et al. reported that the sulfur-containing systems might be tolerant to H_2_S and CO, but would react with H_2_ and C_2_H_2_ [11]. Therefore, the development of a new olefin carrier for separation of propylene/propane is required.

Recently, our group reported that Ag nanoparticles polarized by electron acceptors such as *p*-benzoquinone (*p*-BQ) and 7,7,8,8-tetracyanoquinodimethane (TCNQ) could reversibly interact with olefin molecules such as propylene and ethylene, resulting in the separation performance for olefin/paraffin mixtures [12]. For example, when TCNQ was incorporated into PVP/Ag nanoparticle composites, the selectivity and mixed-gas permeance reached 50 and 3.5 GPU, respectively, for more than 130 h [12]. On the other hand, *p*-BQ was utilized into relatively flexible PEO matrix with Ag nanoparticles, the mixed-gas selectivity was observed as 10 with 15 GPU for more than 240 h [13]. When PEBAX-1657/Ag nanoparticle composites with TCNQ were used as polymer membranes, they showed separation performance such as selectivity of 12.7 and permeance of 10.2 GPU [14].

Furthermore, our group reported that ionic liquid also could induce partial positive charges on the surface of silver nanoparticles, reacting reversibly with olefins such as propylene [15]. Such reversible interactions in ionic liquids were employed for facilitated olefin transport and membranes for separation of olefin/paraffin mixtures [15]. These results suggest the new use for ionic liquids as polarizers for Ag nanoparticles in facilitated olefin transport membranes with *p*-BQ. In particular, the strong interaction between the counteranion of ionic liquid and silver nanoparticles caused the surface of the silver metal to be positively charged and was expected to be utilized as liquid-state membranes for adsorbent materials. Since the ionic liquids could play a role as matrix for the preparation of membranes due to relatively high viscosity, they have been widely utilized in liquid-state membranes. 

In this paper, we reported the stability of ionic liquid/Ag metal composite membrane and suggested the stable membrane for facilitated olefin transport membrane as well as the long-term stable condition for ionic liquids. In particular, the hydrophilic and hydrophobic ionic liquids such as 1-butyl-3-methylimidazolium tetrafluoroborate (BMIM^+^BF_4_^−^), and 1-butyl-3-methylimidazolium hexafluorophosphate (BMIM^+^PF_6_^−^), respectively, were chosen and compared in viewpoint of the interactions between Ag particles and ionic liquids (ILs).

## 2. Materials and Methods

### 2.1. Materials

Silver nanopowder (<100 nm, 99.5%) was purchased from Aldrich Chemical Co (St. Louis, MO, USA). The ionic liquids 1-butyl-3-methylimidazolium tetrafluoroborate (BMIM^+^BF_4_^−^, 98%) and 1-butyl-3-methylimidazolium hexafluorophosphate (BMIM^+^PF_6_^−^, 98%) were purchased from C-TRI Co (Gyeonggi-do, Korea). All the chemicals were used as received. 

### 2.2. Separation Performance

The ILs/Ag composite membranes were prepared by dispersing Ag nanopowder in ILs. The ILs’ weight was fixed at 1g and the amount of the Ag nanopowder was varied. For the fabrication of the separation membranes, the mixed solution was coated onto polyester microporous membrane supports (Osmonics Inc., Minnetonka, MN, USA, pore 0.1 μm) using an RK Control Coater (Model 101, Control Coater RK Print-Coat instruments LTD, UK). The solution consisting of ionic liquid and silver nanoparticles could be maintained as the coating layer as it was on the porous polymer support due to its high viscosity. Gas flow rates or gas permeances were measured with a mass flow meter (MFM). The unit of gas permeance is GPU, where 1 GPU = 1 × 10^−6^ cm^3^ (STP)/(cm^2^ sec cmHg). The mixed gas (50:50 vol % propylene/propane mixture) separation properties of the ILs/Ag composite membranes were evaluated using a gas chromatograph (Hewlett-Packard G1530A, Palo Alto, CA, USA) equipped with a TCD detector and a unibead 2S 60/80 packed column.

### 2.3. Characterization

X-ray photoelectron spectroscopy (XPS) data were acquired using a Perkin-Elmer Physical Electronics PHI 5400 X-ray photoelectron spectrometer (Waltham, Massachusetts, United States). This system was equipped with a Mg X-ray source operated at 300 W (15 kV, 20 mA). The carbon (C 1s) line at 285.0 eV was used as the reference in our determinations of the binding energies of the silver.

## 3. Results

### 3.1. Performance in Separation of Propylene/Propane Mixtures

**Figure 1:**
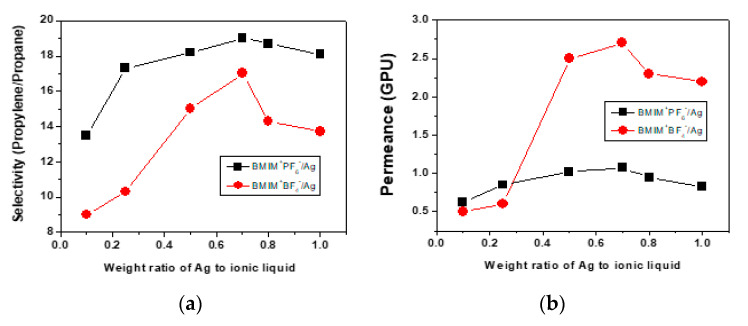
Separation performance: (**a**) mixed gas selectivity and (**b**) total permeance of BMIM^+^BF_4_^−^ and BMIM^+^PF_6_^−^ membranes with various weight ratios of silver nanoparticles.

The separation of propylene/propane mixtures using BMIM^+^BF_4_^−^ and BMIM^+^PF_6_^−^ membranes was evaluated with varying concentrations of the silver nanoparticles. The BMIM^+^BF_4_^−^ and BMIM^+^PF_6_^−^ membranes without silver nanoparticles exhibited low gas permeation and no separation of the propylene/propane mixtures; mixed gas permeance is ca. 0.1 GPU (1 GPU = 1 × 10^−6^ cm^3^(STP) cm^−2^ s^−1^cmHg^−1^) and the selectivity of propylene/propane is nearly unity. Figure 1 shows the selectivity and total permeance of propylene over propane through BMIM^+^BF_4_^−^ and BMIM^+^PF_6_^−^ membranes containing silver nanoparticles. The presence of silver nanoparticles in the BMIM^+^BF_4_^−^ and BMIM^+^PF_6_^−^ membranes resulted in an increase in both the selectivity and the permeance. It was thought that the interactions between the silver nanoparticle and the counteranion of the ionic liquid would cause the silver nanoparticles to be partially polarized, resulting in the facilitated olefin transport. The selectivity of propylene/propane and the mixed gas permeance in BMIM^+^BF_4_^−^/Ag membrane increased to 17 and 2.7 GPU, respectively. However, at weight ratios higher than 0.7, the propylene permeance decreased with the increasing amount of silver metal, presumably due to the aggregation of the nanoparticles and consequently the loss of the carrier activity. Furthermore, the aggregation of Ag nanoparticles could play a role as barriers for gas transport, resulting in the decrease in gas permeance.

On the other hand, for the BMIM^+^PF_6_^−^/Ag membrane, the selectivity of propylene/propane and the mixed gas permeance increased to 19 and 1.1 GPU, respectively, as shown in Table 1. Interestingly, BMIM^+^PF_6_^−^/Ag membrane showed the higher selectivity than BMIM^+^BF_4_^−^/Ag membrane, even at a low silver concentration. It was thought that the interaction between PF_6_^−^ and silver nanoparticle was stronger than that between BF_4_^−^ and silver nanoparticle. Since the surface polarity of Ag nanoparticles increased, the reversible interaction with olefin molecules was enhanced, resulting in the increase in selectivity for propylene/propane mixtures. On the other hand, low permeance of BMIM^+^PF_6_^−^/Ag membrane was attributable to the hydrophobicity of BMIM^+^PF_6_^−^, capturing the propylene and propane molecules in ionic liquid. Furthermore, the viscosity of BMIM^+^BF_4_^−^ was 0.0325 Pa · s, while that of BMIM^+^PF_6_^−^ was 0.05 Pa · s, as shown in Table 2. Higher viscosity of BMIM^+^PF_6_^−^ indicated the relatively higher interactions between cations and counteranions, resulting in the diminishment of mixed-gas permeance. Thus, the hydrophobic properties and higher viscosity played a role as factors for low mixed-gas permeance.

**Figure 2:**
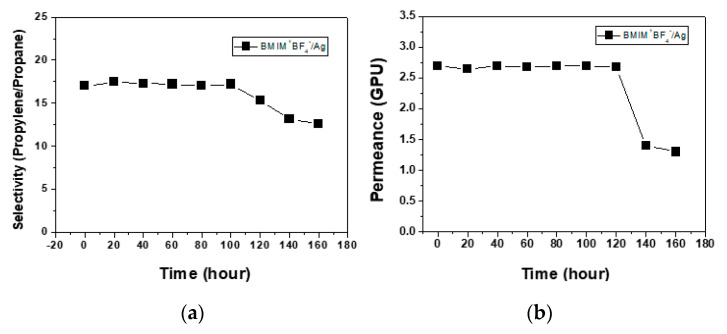
Separation performance: (**a**) mixed gas selectivity and (**b**) total permeance of 1/0.7 BMIM^+^BF_4_^−^/Ag membranes as a function of time (40 psig and 20 °C).

**Figure 3:**
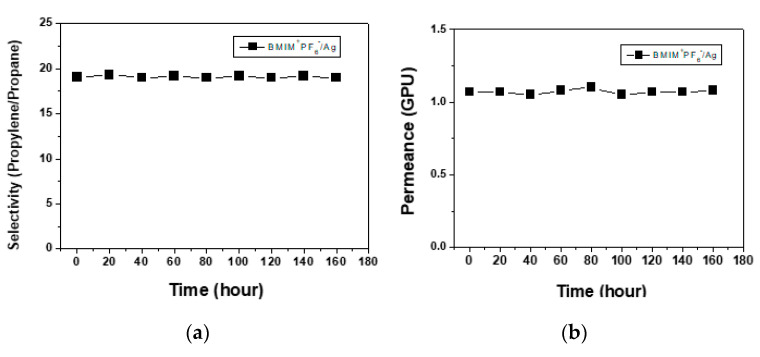
Separation performance: (**a**) mixed gas selectivity and (**b**) total permeance of 1/0.7 BMIM^+^PF_6_^−^/Ag membranes as a function of time (40 psig and 20 °C).

The stability of BMIM^+^BF_4_^−^ and BMIM^+^PF_6_^−^/Ag composite membranes was also tested by measuring the separation performances of propylene/propane mixtures for up to 160 h. Figure 2 and Figure 3 show the selectivity and mixed gas permeance with respect to propylene/propane of the BMIM^+^BF_4_^−^ and BMIM^+^PF_6_^−^ membranes containing the silver nanoparticles, respectively. The weight ratio of silver nanoparticles to ionic liquid was fixed at 1/0.7. The BMIM^+^BF_4_^−^/Ag composite membrane showed stability up to 100 h, but decreased after 100 h, possibly due to the particle aggregation in the membrane. It was thought that these aggregation phenomena of Ag nanoparticles in BMIM^+^BF_4_^−^ was attributable to the relatively weak interaction between the surface of the metal and the hydrophilic ionic liquid since the utilized metal had hydrophobic properties.

On the other hand, the selectivity and the permeance of BMIM^+^PF_6_^−^/Ag composite membrane were nearly invariant for the duration of the experiment up to 160 h, which suggested that particles aggregation did not happen. It was supposed that the interaction between the hydrophobic BMIM^+^PF_6_^−^ and silver nanoparticles was stronger than that between the hydrophilic BMIM^+^BF_4_^−^ and silver nanoparticles. These favorable interactions between BMIM^+^BF_4_^−^ and silver nanoparticles caused the metal to be maintained as non-aggregated state even under gas transport with time. From these results, the hydrophobic properties in ionic liquid-based composite membranes were more desirable for long-term stable membranes.

These experimental results suggest that the ionic liquids could cause a favorable interaction between the silver particles and olefin, resulting in facilitated olefin transport and improved separation performance for olefin/paraffin mixtures. In particular, the hydrophobic BMIM^+^PF_6_^−^ ionic liquid was more desirable for the long-term stable membrane than hydrophilic BMIM^+^BF_4_^−^, even though BMIM^+^BF_4_^−^/Ag membranes showed higher mixed-gas permeance. These stable separation performances were related to the sustainable polarity of silver nanoparticles. Thus, the stability of polarity in silver particles was characterized in the following sections in terms of the changes in binding energy, as examined by spectroscopy.

### 3.2. Change of Binding Energies

**Figure 4:**
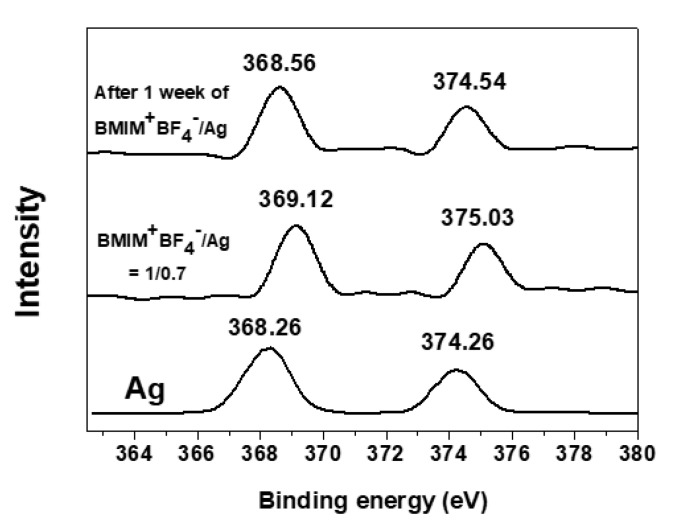
Binding energy of Ag in 1/0.7 BMIM^+^BF_4_^−^/Ag composites after preparation and after 1 week.

**Scheme 1:**
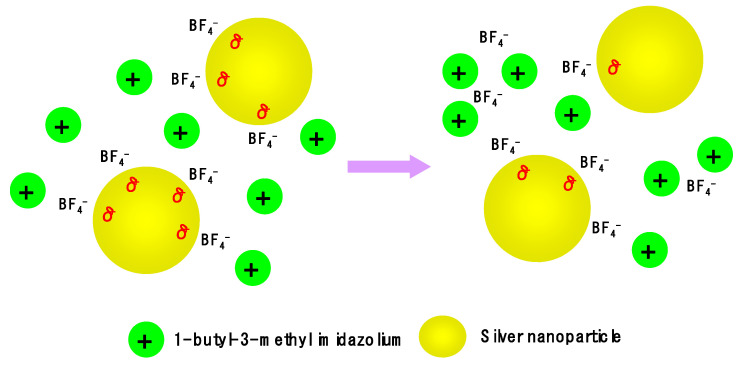
Ag nanoparticles in ionic liquid BMIMBF_4_ with time.

X-ray photoelectron spectroscopy (XPS) was used to observe the change in the chemical environment with time around the silver nanoparticles in ionic liquid/Ag metal composite. It was well known that the d_5/2_ orbital of Ag was observed at 368.26 eV and could be shifted by a change in electron density in the metal [15]. Interestingly, the binding energy of the d_5/2_ orbital of the silver particle in the 1/0.7 BMIM^+^BF_4_^−^/Ag metal composite system increased gradually from 368.26 to 369.12 eV, as shown in Figure 4. This indicated that the binding energy of the valence electrons in the silver atoms increased due to the interactions between the silver atoms and BMIM^+^BF_4_^−^, and that the surface of the silver nanoparticles was partially positively charged [15]. These results indicate that the ionic liquid could change the state of electron density in the surface of Ag metal, resulting in the positive polarity on metal [15]. However, after 1 week, the binding energy of the d_5/2_ orbital of the silver particle decreased from 369.12 to 368.56 eV due to the phase separation, resulting in the deterioration of separation performance. As shown in Scheme 1, the Ag nanoparticles in the ionic liquid BMIM^+^BF_4_^−^ were stabilized and positively polarized, showing the facilitated propylene transport. However, the Ag nanoparticles were aggregated with time due to the relatively weak interaction between the surface of the metal and the ionic liquid, resulting in poor long-term stability.

**Figure 5:**
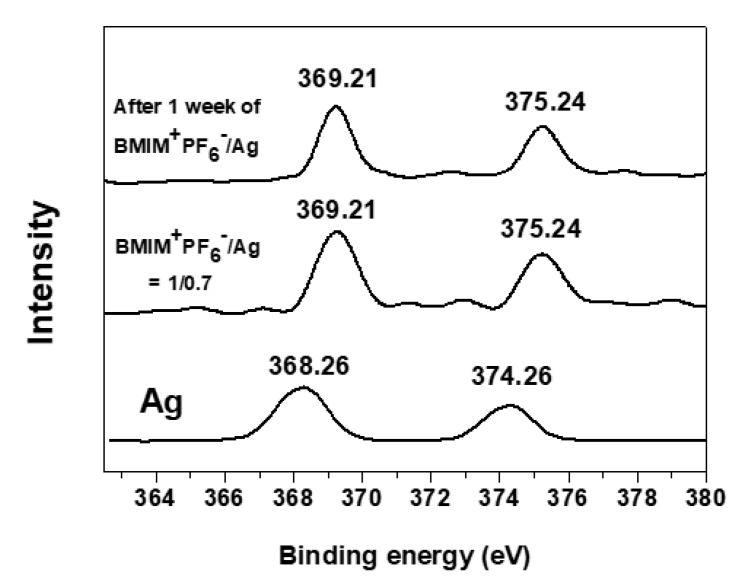
Binding energy of Ag in 1/0.7 BMIM^+^PF_6_^−^/Ag composites after preparation and after 1 week.

**Scheme 2:**
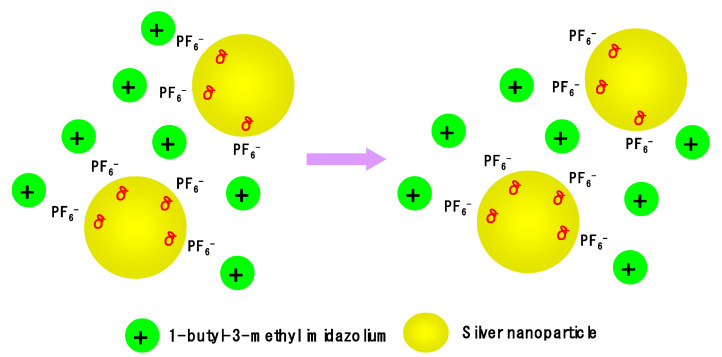
Ag nanoparticles in ionic liquid BMIMPF_6_ with time.

On the other hand, the binding energy of the d_5/2_ orbital of the silver particle in the 1/0.7 BMIM^+^PF_6_^−^/Ag metal composite system increased gradually from 368.26 to 369.21 eV, as shown in Figure 5. The increased binding energy of Ag metal indicated the positive polarity of surface, resulting in the facilitated propylene transport. Surprisingly, the binding energy remained constant even after 1 week, indicating that the phase was not separated, as shown in Scheme 2, consistent with long-term stable separation performance.

Therefore, it can be concluded that the hydrophobic ionic liquid was more desirable than the hydrophilic ionic liquid for facilitated olefin transport membrane from the viewpoint of long-term stability.

## 4. Conclusions

In summary, we succeeded in preparing for the long-term stable composites consisting of BMIM^+^PF_6_^−^ and Ag metal for liquid-based membranes. BMIM^+^PF_6_^−^/Ag nanocomposite membranes showed long-term stability for more than 160 h, while the deterioration of separation performance in BMIM^+^BF_4_^−^/Ag nanocomposite membranes was observed. In fact, the binding energy of Ag metal in BMIM^+^PF_6_^−^ remained constant even after 1 week due to a strong interaction between the surface of Ag and ionic liquids. The separation performance and the XPS data indicated that the hydrophobic BMIM^+^PF_6_^−^/Ag metal composite membrane was more stable than hydrophilic BMIM^+^BF_4_^−^/Ag metal composite membrane, since the surface of silver metal was hydrophobic, resulting in the long-term stable attraction between ionic liquid and surface of nanoparticles. From these results, we could suggest the crucial factor for long-term stability in liquid-based membranes.

## Figures and Tables

**Table 1:** Separation performance of BMIM^+^BF_4_^−^/Ag and BMIM^+^PF_6_^−^/Ag composite membranes.

	Mixed-Gas Selectivity	Permeance (GPU)
BMIM^+^BF_4_^−^/Ag	17	2.7
BMIM^+^PF_6_^−^/Ag	19	1.1

**Table 2:** Properties of BMIM^+^BF_4_^-^ and BMIM^+^PF_6_^-.^

	BMIM^+^BF_4_^−^	BMIM^+^PF_6_^−^
Chemical structure	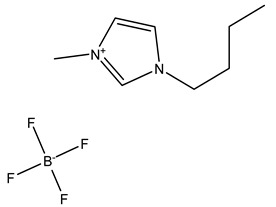	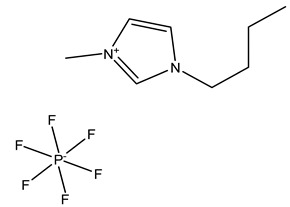
Melting point	−80 °C	−8 °C
Solubility in water	soluble	insoluble
Viscosity (Pa · s)	0.0325	0.05
Henry’s constants (mol·kg^−1·^kPa^−1^) of C_3_H_6_ (298 K)	5.7·10^−4^	-
